# Atlanta Society of Medicine

**Published:** 1885-12-20

**Authors:** 


### ATLANTA SOCIETY OF MEDICINE.

At the last annual election of this Society, held on the 1st instant, the following officers were elected :

President—Dr. J. Donald Wilson.
Vice President—Dr. N. O. Harris.
Secretary—Dr. Virgil O Hardon.
Treasurer—Dr. W. B. Parks.
Corresponding Secretary and Librarian—Dr. W. G. Elkin.
Reporters - Drs. Henry Wile, F. W. McRae, E. van Goidtsnoven.
Censor—Dr. C. C. Greene.

Dr. George H. Noble presented and read a very interesting paper on Cerebral Anæmia as a Cause of Asphyxia in the New-born. He reported two cases that show a new departure from the old routine mode of treating the still-born. He conceived anæmia of the brain, or a condition of syncope, to be the cause of the asphyxia, and placed the babies in a perpendicular position with their heads downwards, that the blood might gravitate to the brain and enable that organ to perform its proper functions. In the first case it was due, he thought, to the feeble heart's action, as the child was repeatedly changed, from the horizontal to the inverted position, and each time that it was removed from the latter all attempts at respiration ceased ; but, on the contrary, they were induced, whenever the babe was placed head downward. The second case was twice saved by this means, for, after having established respiration, the nurse carelessly placed him in a sitting position to put on his shirt, which resulted in "secondary asphyxia." The cause of the suspended animation in this case was, doubtless, due to an anæmic conditon of the brain from pressure in birth, as there was very great overlapping of the cranial bones, and as the heart's action was, at first, strong and forcible, but afterward grew weak. The secondary aspyxia was due to the weak heart, as it could not pump the blood to the brain with the child in the sitting position. All other methods of resuscitation entirely failed in these cases, which was the cause of the doctor's resorting to this treatment.

He claimed for it certain advantages : "1. It enables the practitioner to employ other methods of resuscitation in conjunction with it. 2. It is totally void of violence—a matter worthy of consideration. 3. It induces the escape of such fluids as may be in the air-passages. 4. It substitutes the pure blood of the inferior vena cava for the venous blood of the superior vena cava, where there is a circulation through the foramen ovale. 5. It gravitates blood to the lungs, which, by distending the blood vessels, causes stimulation of respiration."

